# Key Role of ROS in the Process of 15-Lipoxygenase/15-Hydroxyeicosatetraenoiccid-Induced Pulmonary Vascular Remodeling in Hypoxia Pulmonary Hypertension

**DOI:** 10.1371/journal.pone.0149164

**Published:** 2016-02-12

**Authors:** Qian Li, Min Mao, Yanli Qiu, Gaofeng Liu, Tingting Sheng, Xiufeng Yu, Shuang Wang, Daling Zhu

**Affiliations:** 1 Department of Pharmaceutical Analysis, College of Pharmacy, Harbin Medical University, Harbin, Heilongjiang Province, China; 2 Biological Sciences, Purdue University, West Lafayette, Indiana, United States of America; 3 Department of Pathophysiology, Harbin Medical University-Daqing, Daqing, Heilongjiang Province, China; 4 Bio-pharmaceutical Key Laboratory of Harbin, Harbin Medical University, Harbin, Heilongjiang Province, China; 5 Department of Pharmacy, the Second Affiliated Hospital, Harbin Medical University, Harbin, Heilongjiang Province, China; Indiana University, UNITED STATES

## Abstract

We previously reported that 15-lipoxygenase (15-LO) and its metabolite 15-hydroxyeicosatetraenoic acid (15-HETE) were up-regulated in pulmonary arterial cells from both pulmonary artery hypertension patients and hypoxic rats and that these factors mediated the progression of pulmonary hypertension (PH) by affecting the proliferation and apoptosis of pulmonary arterial (PA) cells. However, the underlying mechanisms of the remodeling induced by 15-HETE have remained unclear. As reactive oxygen species (ROS) and 15-LO are both induced by hypoxia, it is possible that ROS are involved in the events of hypoxia-induced 15-LO expression that lead to PH. We employed immunohistochemistry, tube formation assays, bromodeoxyuridine (BrdU) incorporation assays, and cell cycle analyses to explore the role of ROS in the process of 15-HETE-mediated hypoxic pulmonary hypertension (HPH). We found that exogenous 15-HETE facilitated the generation of ROS and that this effect was mainly localized to mitochondria. In particular, the mitochondrial electron transport chain and nicotinamide-adenine dinucleotide phosphate oxidase 4 (Nox4) were responsible for the significant 15-HETE-stimulated increase in ROS production. Moreover, ROS induced by 15-HETE stimulated endothelial cell (EC) migration and promoted pulmonary artery smooth muscle cell (PASMC) proliferation under hypoxia via the p38 MAPK pathway. These results indicated that 15-HETE-regulated ROS mediated hypoxia-induced pulmonary vascular remodeling (PVR) via the p38 MAPK pathway.

## Introduction

Hypoxic pulmonary hypertension (HPH) is a progressive disease characterized by elevated pulmonary vascular resistance, which leads to right ventricular (RV) failure and significantly increases morbidity and mortality [1, 2]. The pathological process associated with pulmonary hypertension (PH) is the striking structural remodeling of the pulmonary arteries (PAs), particularly in the media [3]. However, it remains unclear what drives the abnormal pulmonary vascular development and structural remodeling in HPH.

It has been reported that increased oxidative stress augments HPH [4], whereas reduced oxidative stress can reverse it [5]. Following the recognition that reactive oxygen species (ROS) serve as important signaling molecules, multiple lines of evidence have shown that ROS are released from pulmonary artery endothelial cells (PAECs) and subsequently stimulate vascular smooth muscle cell (SMC) proliferation, which, in turn, results in pulmonary vascular remodeling (PVR) under hypoxic conditions [6–9]. How ROS are generated in hypoxic PAs is not well defined. Vascular NADPH oxidases have received increasing attention as important sources of the ROS that contribute to HPH. Nox4, one subtype with seven isoforms in the NADPH oxidase family, is extensively distributed in various cell types, including vascular endothelial cells (ECs) and smooth muscle cells (SMCs). In particular, Nox4 has been shown to be a crucial player in EC migration and SMC proliferation [10–12].

A large number of scientific studies have highlighted the importance of the p38 mitogen-activated protein kinase (MAPK) pathway in numerous cellular processes, including cell proliferation, gene expression, adhesion, differentiation, senescence, and apoptosis. Emerging evidence indicates that activation of the p38 MAPK pathway may also mediate hypoxia-induced PA endothelial dysfunction and lead to EC proliferation/apoptosis. However, the mechanism responsible for the activation of p38 MAPK by hypoxia and whether p38 MAPK is involved in hypoxia-induced vascular remodeling during HPH remain largely unclear.

Our previous study demonstrated that 15-lipoxygenase (15-LO) was up-regulated in PAECs and pulmonary artery smooth muscle cells (PASMCs) under hypoxic conditions and that its main metabolite, 15-hydroxyeicosatetraenoic acid (15-HETE), is an important mediator of HPH regulation, including pulmonary vasoconstriction, vascular medial hypertrophy, and remodeling [13–16]. We also demonstrated that hypoxia-enhanced 15-HETE stimulated cell cycle progression, promoted PASMC proliferation, induced pulmonary vascular medial hypertrophy and intimal endothelial cell migration, and ultimately led to PVR [17–19]. However, the underlying mechanisms for the remodeling regulated by 15-HETE under hypoxia have not been fully clarified. We hypothesized that 15-HETE up-regulated the expression of Nox4 in PAECs, thereby inducing ROS production, promoting EC migration and SMC proliferation, and ultimately leading to PVR and PH. Our results in the present work show that 15-HETE enhanced ROS production, promoted vascular remodeling, and exerted these effects, at least in part, via the p38 MAPK pathway.

## Materials and Methods

### Ethics Statement

The work was approved by the Harbin Medical University Ethical Committee for Use of Human Samples. All experimental procedures in animals were carried out in accordance with guidelines for the Care and Use of Laboratory Animals approved by the Institutional Animal Care and Use Committee and were conducted in compliance with the NIH guidelines. The study protocol on the Ethics of Animal was approved by the Experiments Committee of Harbin Medical University (Permit Number: 2010–0006). All surgery was carried out under sodium pentobarbital anesthesia, and all efforts were made to minimize pain.

### Animals and lung tissue preparation

All animal protocols were approved by the Institutional Animal Care and Use Committee. Adult female Wistar rats with a mean weight of 200 g were purchased from the Harbin Medical University Experimental Animal Center. Rats were randomly assigned to 9 days in a normal or hypoxic environment with fractional inspired oxygen at 0.21 and 0.12, respectively. Normoxic rats were kept in the same room adjacent to the hypoxic chamber. At the same time, one group of rats was administered with nordihydroguaiaretic acid (NDGA, inhibitor of 15-LO, 650 mg/kg body weight, orally, once daily) beginning 2 days before hypoxia and continuing until euthanasia (the 10th day after hypoxia), while another group of rats was injected with monocrotaline (MCT, 60 mg/kg). At the end of the 9th day of exposure, we anesthetized each rat with a pentobarbital injection (120 mg/kg, i.p.), opened the thoraxes and quickly detached the lungs, which were further processed for immunocytochemistry. At the end of the 9-day exposure period, we anesthetized and dissected each rat as previously described [20].

### Histology and immunohistochemistry

The rat lung tissues were immobilized in 4% paraformaldehyde for 48 hours and then dehydrated and embedded in paraffin wax. For H&E and Masson staining, the paraffin tissue was sliced into 5-μm slices and stained with the dye. Human lung samples were obtained as previously described [21]. For immunohistochemistry, the 5-μm paraffin tissue slices were dewaxed and restored before overnight incubation with anti-Nox4 antibodies. The primary antibodies were removed by washing with PBST, and the tissues were incubated with secondary IgG antibodies before staining with 3, 3-diaminobenzidine (DAB) and restaining with hematoxylin. The immunoreactivity of Nox4 in the vascular tunnel was visualized with high-resolution images of individual vessel walls using image analysis and a color-recognition algorithm of Image-Pro Plus 6.0, as previously described [22, 23].

### Cell culture

PAECs and PASMCs were respectively prepared from aortal arteries and PAs collected from calf lungs obtained from a local slaughterhouse. This protocol was approved by the Harbin Medical University Ethical Committee of Laboratory Animals. The arteries were gently slit, and the innermost layer was scraped with a surgical blade to obtain endothelial cells. The arteries were then cut into small pieces, and the smooth muscle layer was affixed to the culture dish to allow the smooth muscle cells to climb out for a period of 2 hours. The arterial fractions were covered with Dulbecco's modified Eagle's medium (DMEM) supplemented with 20% fetal bovine serum (FBS). The tissue fractions were then lifted out of the medium, and the adherent SMCs were allowed to proliferate. Using antibodies to CD31 (Santa Cruz Biotechnology), the purity and identity of ECs were confirmed by positive immunofluorescence staining. The purity and identity of SMCs were determined by immunocytochemical staining with antibodies against smooth muscle α-actin as previously described [22, 24, 25].

### Immunofluorescence

Cells were pretreated with apocynin (APO, Nox4 inhibitors, 10 μmol/L) or rotenone (RE, a mitochondrial inhibitor, 2 μmol/L) for 30 minutes and subsequently treated with 15-HETE for 2 hours. The culture medium was then removed, and ROS detection reagents CM-H2DCFDA (10 μmol/L) or MitoSOX (5 μmol/L) were applied for the required durations in the dark. The cells were then washed three times with buffer solution. Measurements were made with a fluorescence microscope (excitation, 488 nm; emission, 585 nm) and analyzed using MetaMorph software (Molecular Devices). The data are reported as the fluorescence intensity.

### Immunocytochemistry

The PASMCs were cultured on a poly-L-lysine-coated cover glass (15-mm diameter) and washed with PBS, followed by fixation with 4% paraformaldehyde at room temperature for 15 minutes. After permeabilizing with 0.01% Triton X-100 for 10 min, the cells were blocked with 3% normal bovine serum at 37°C for 30 min and then incubated with anti-α-tubulin primary antibodies (1:50) in PBS at 4°C overnight. After washing with PBS, the cells were incubated with FITC-conjugated secondary antibodies (1:100) diluted in PBS as well as Hoechst at 37°C for 2 hours, protected from light. The cover glass was then mounted and analyzed with a confocal laser-scanning microscope (CLSM) as previously described [21, 25]. The images were merged using the CLSM. The data are reported as the fluorescence intensity.

### Western blot analysis

Pretreatment was performed with 15-HETE (1 μmol/L), cinnamyl 3, 4-dihydroxy-[alpha]-cyanocinnamate (CDC, inhibitor of 15-LO, 5 μmol/L), NDGA (30 μmol/L), APO (10 μmol/L), N-acetyl-l-cysteine (NAC, ROS scavenger, 25 μmol/L) plus 15-HETE, 4-(4-fluorophenyl)-2-(4-methylsulfinylphenyl)-5-(4-pyridyl)-1H-imidazole (SB203580, p38 MAPK inhibitors, 10 μmol/L) plus 15-HETE or H_2_O_2_ (50 μmol/L) plus 15-HETE in DMEM with 5% FBS for 24 hours. The cells were then washed with PBS three times, and lysates were prepared with RIPA lysis buffer. To isolate mitochondrial proteins, after incubation for 24 hours, the mitochondria were isolated from cells using a Cell Mitochondria Isolation Kit, and then RIPA lysis buffer was added to extract the mitochondrial proteins. The protein concentrations in the supernatants were measured with the bicinchoninic acid protein assay (Pierce, Rockford, IL), based on bovine serum albumin (BSA) standards. The proteins were separated by 10% SDS-PAGE and electrotransferred to nitrocellulose membrane (Millipore, USA). The membranes were blocked with 5% milk in TBST for 3 hours at 4°C, followed by incubation with primary antibodies against Nox4 and proliferating cell nuclear antigen (PCNA) (1:200 in 5% BSA) at 4°C overnight. The membranes were subsequently incubated with horseradish peroxidase-conjugated secondary antibodies (1:10,000, Santa Cruz Biotechnology) for 30 minutes and then with enhanced chemiluminescence reagents, as previously described [22, 26].

### Real-time quantitative PCR

Using TRIzol reagent per the manufacturer’s instructions, total RNA was extracted from cultured cells after treatment with the indicated reagents for 24 hours. The RNA from each sample was reverse-transcribed using the Superscript First-Stand cDNA Synthesis Kit (Invitrogen CA, USA). Gene-specific primers were designed from coding regions similar to those obtained from the GenBank^™^ database. β-actin was used as an internal control. The specific primer sequences were devised and synthesized as follows (Shinegene Co., Shanghai): β-actin (NM_173979): sense, *5’-TTAGCTGCGTTACACCCTT-3’*, antisense, *5’-GTCACCTTCACCGTTCCA-3’*; Nox4 (XM_002699032): sense, *5’-TTCTGGACCTTTGTGCCT-3’*, antisense, *5’- CTTTGACCATTCGGATTT-3’*, as previously described [15, 27]. Quantitative RT-PCR was performed with SYBR Green I using an ABI Prism 7300 sequence detection system (Applied Biosystems, Foster City, CA).

### RNA interference of 15-LO_1_ and 15-LO_2_

To suppress the expression of 15-LO_1_ and 15-LO_2_, PAECs were transfected with small interfering RNAs designed and synthesized by GenePharma (Shanghai, China). Non-targeted control siRNA (siNC) was used as a negative control as previously described [28, 29]. The sequences used for silencing 15-LO_1_ (NM_174501) and 15-LO_2_ (NM_001205703) were *5’-GACGGGUAAUUCUGAAUATTUAUUCAGAA-3’* and *5’-CCGCACCAAUGUCAUCAAUTTAUUGAUGAC-3’*, respectively. The negative control had the sequence *5’-UUCUCCGAACGUGUCACGUTTACG-3’*. Transfection was performed at a concentration of 180 nmol/L, with 1.5 μg siRNA for 15-LO_1_, 15-LO_2_, and the negative control mixed with 7.5 μL of X-treme siRNA transfection reagent (an optimized lipid-based reagent that forms a complex with short interfering RNA (siRNA) and mixtures of siRNA and plasmid DNA, in order to introduce siRNA into animal cells) and dripped onto the cells. Four hours later, the medium was aspirated and replaced with DMEM containing 20% FBS. Cellular proteins were harvested after 24 hours.

### BrdU incorporation assay

After pretreatment with 15-HETE (1 μmol/L), CDC (5 μmol/L), NDGA (30 μmol/L), NAC (25 μmol/L), SB203580 (10 μmol/L) plus 15-HETE or H_2_O_2_ (50 μmol/L) plus 15-HETE in DMEM supplemented with 5% FBS for 24 hours, cultured SMCs in 96-well culture plates were incubated with 5-BrdU labeling solution for approximately 2 hours. The labeling medium was then aspirated, and FixDenat was added to the cells for 30 minutes at 37°C. The cells were then removed from the FixDenat solution and added to anti-BrdU-POD solution for 90 minutes. The antibody conjugate was removed by rinsing with wash solution, and the cells were placed in substrate solution. Data were measured by spectrophotometric absorbance at 390 nm, as previously described [15, 30].

### Scratch-wound assay

PAECs were cultivated in a 6-well culture plate and scratched with pipette tips. The cells were then pretreated as in the BrdU incorporation assay before being photographed at 0 and 8 hours, as previously described [31, 32]. The rate of migration was measured with Image Pro-Plus 6.0.

### Tube formation assay

A 96-well culture plate was covered with growth factor-reduced Matrigel for 30 minutes at 37°C and allowed to solidify prior to the addition of PAECs. Next, 15-HETE, SB203580 (10 μmol/L) plus 15-HETE, or SB203580 (10 μmol/L) and H_2_O_2_ (50 μmol/L) plus 15-HETE were added to the medium of different wells. Photographs were taken once the tubes were formed. Tube length was calculated using Image Pro-Plus 6.0 as previously described [27, 31].

### Flow cytometry

Different reagents were added to cultured cells (as in the BrdU incorporation assay) at different concentrations for 24 hours. The cells were washed with PBS and then fixed with 75% ethanol for another 24 hours at 4°C. After incubation in 0.5 ml PBS containing 10 μg/ml RNase A and 100 μg/ml PI for 30 minutes at 37°C in the dark, DNA fluorescence was measured in the immobilized cell samples using a BD FACSCalibur Flow Cytometer (Bedford, MA) as previously described [25, 33]. The DNA content at each phase of the cell cycle was recorded.

### Statistical analysis

All values are expressed as the mean ± SEM. Statistical analysis was performed using Student’s t-tests or one-way analysis of variance (ANOVA) followed by Dunnett's test where appropriate. A value of *p*<0.05 was considered statistically significant.

## Results

### Hypoxia increase the production of ROS through the 15-LO/15-HETE pathway

**Fig 1:**
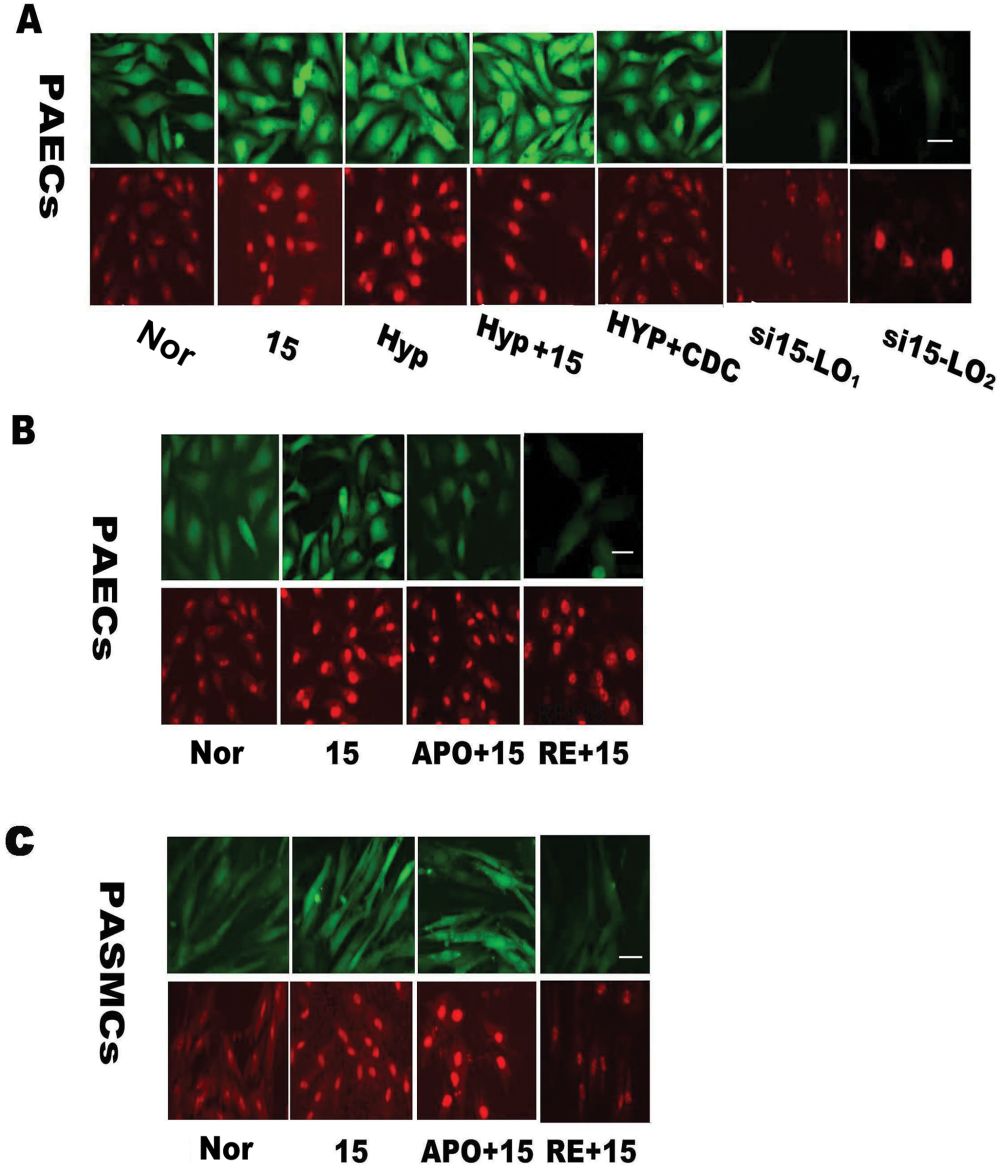
Immunofluorescence is employed to evaluate ROS production in cells (green) and mitochondria (red). **(A)** PAECs were pretreated with CDC (inhibitor of 15-LO) or si15-LO and then exposed to 15-HETE and CM-H2DCFDA or MitoSOX to measure the effect of 15-HETE on the production of ROS. To locate the target of 15-HETE, PAECs **(B)** and PASMCs **(C)** were incubated with APO (apocynin, an NADPH oxidase inhibitor) or RE (rotenone, a mitochondria inhibitor) prior to treatment with 15-HETE. ROS were assessed by CLSM after treatment with CM-H_2_DCFDA and MitoSOX. Green, cellular ROS; red, mitochondrial ROS. Scale bars equal 100 μm, Nor, normoxia; Hyp, hypoxia; 15, 15-HETE.

To evaluate the effect of hypoxia on the production of ROS, we labeled PAECs and PASMCs with CM-H2DCFDA or MitoSOX molecular probes. These assays demonstrated that ROS were increased in PAECs and their mitochondria under hypoxic conditions. Cinnamyl 3, 4-dihydroxy-[alpha]-cyanocinnamate (CDC) (an inhibitor of 15-LO) alleviated the effect of hypoxia on ROS production (in Fig 1A, CM-H2DCFDA is green, and MitoSOX is red). As CDC may cause non-specific inhibition of 15-LO, we also employed siRNA interference to specifically inhibit the expression of 15-LO in PAECs. Si15-LO_1_ and si15-LO_2_ also reduced the hypoxia-induced ROS production in endothelial cells and PAEC mitochondria. These data indicated that the 15-LO/15-HETE pathway may contribute to the process by which hypoxia regulates ROS production.

It is well known that ROS can originate from several different reaction steps of the mitochondrial electron transport chain, especially in the reactions involving xanthine oxidase, nicotinamide adenine dinucleotide phosphate (NADPH) oxidases (Nox enzymes), and other enzymes [34, 35]. However, relatively little is known about the main cellular sources of ROS that contribute to the process of 15-HETE-mediated hypoxic PVR. We hypothesized that the ROS induced by 15-HETE are mainly generated by NADPH oxidases and mitochondria. To test this hypothesis, we treated the PAECs with APO (apocynin, a Nox4 inhibitor) or RE (rotenone, a mitochondrial inhibitor) to determine the cellular source of ROS. We found that both APO and RE decreased the cellular and mitochondrial ROS in PAECs (Fig 1B and 1C). However, different results were observed in PASMCs and their mitochondria, where RE but not APO treatment blocked the increased production of ROS triggered by 15-HETE. These data indicated that the main sources of ROS in PAECs were NADPH oxidase (APO) and mitochondria, but mitochondria played a predominant role in ROS production in PASMCs. This finding implies the existence of distinct regulatory mechanisms in the vascular endothelium and the smooth muscle layer. Taken together, these results demonstrated that the 15-LO/15-HETE pathway may be involved in the process by which hypoxia induces ROS production, with the main cellular sources of ROS in PAECs being the Nox4 pathway and the mitochondrial electron transport chain.

### Nox4 expression is up-regulated in PH patients and PH rat models in a 15-LO/15-HETE-dependent manner

**Fig 2:**
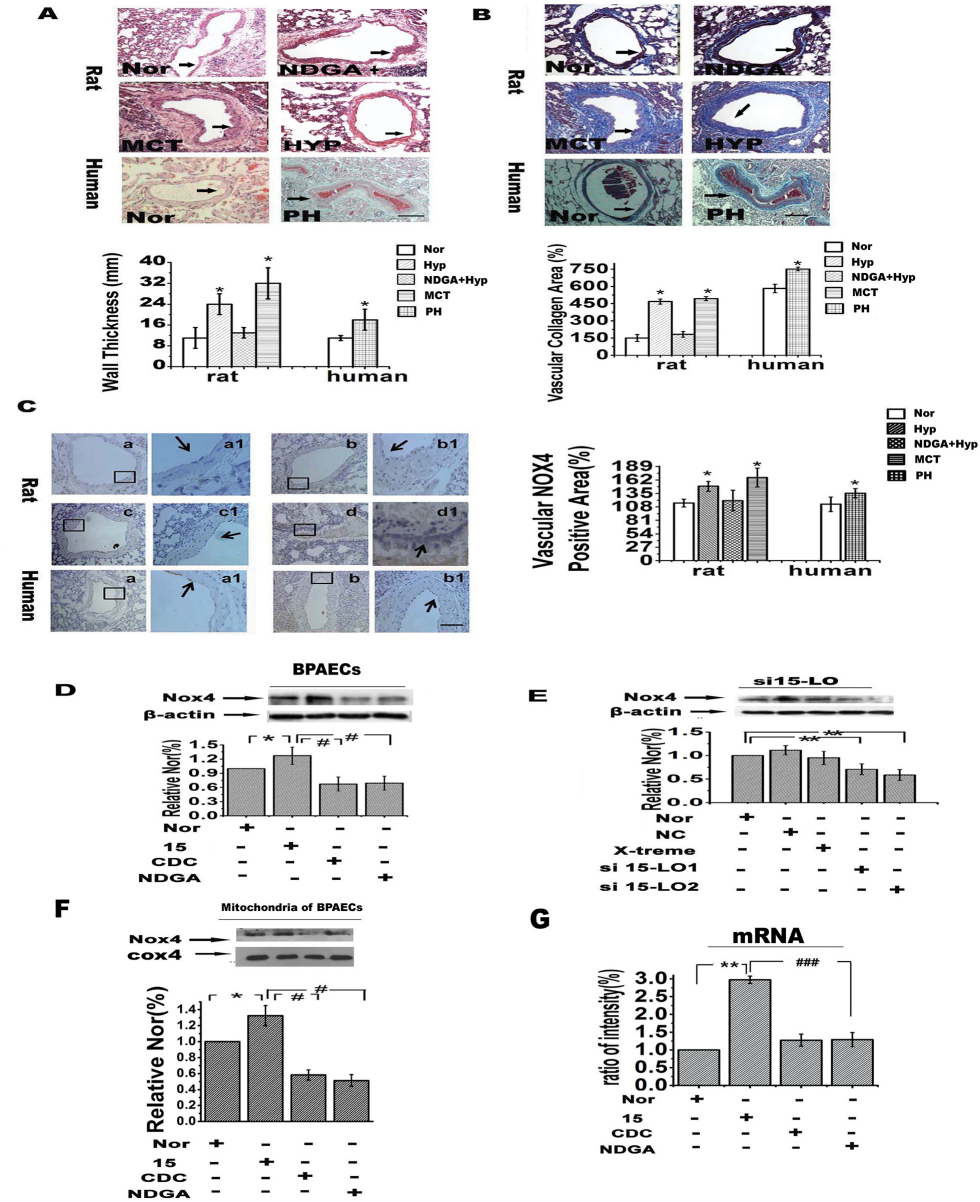
H&E and Masson staining and Nox4 expression in human and rat pulmonary vessels. Histological analysis of rat (n = 6) and human lung tissues (n = 3): (**A**) H&E staining; (**B)** collagen deposition; scale bars equal 100 μm. (**C**) Nox4 expression in PAs from rat (n = 6) and human lungs (n = 3) was evaluated by immunohistochemistry. Data reflect quantitative analyses of positive staining per vascular area. Framed areas in **a** through **d** are shown at high magnification in **a1** through **d1**. Scale bars in **Ca1** through **Cd1** equal 20 μm, and all others are 50 μm. Groups of rat lung tissues: a: Nor; b: Hyp; c: NADC + Hyp; d: MCT. Groups of human lung tissues; a: Nor; b: PH. After incubation with CDC, NDGA (an inhibitor of 15-LO) or si15-LO (**E**), Nox4 protein (**D**) and mRNA (**G**) expression levels in PAECs were evaluated by western blot and real-time PCR. Nox4 protein levels in mitochondria (**F**) were also examined (n = 4). All of the values reflect the means ± SEM; *p<0.05, **p<0.01 versus normal; **#***p*<0.05, ##*p*<0.01 versus Hyp+15. Nor, normal; Hyp, hypoxia; MCT, monocrotaline; PH, pulmonary hypertension; X-treme, siRNA Transfection Reagent.

Morphometric analysis of the pulmonary vasculature with hematoxylin and eosin (H&E) and Masson staining demonstrated thicker pulmonary vascular walls and increased collagen deposition in lung tissue sections of human PH compared with normal control tissue (Fig 2A and 2B). These results were similar to the pathological changes observed in the PAs of hypoxic rats exposed to hypoxia for 9 days and those of MCT-induced PAH rats injected with monocrotaline. In these animals, PA wall thickness (Fig 2A) and collagen deposition (Fig 2B) were significantly increased compared with normoxic rats. This increase was partially blocked by treatment with NDGA (nordihydroguaiaretic acid, a 15-LO inhibitor), consistent with our previous reports. More importantly, the expression of Nox4 was up-regulated in human PH tissue compared with normal tissue samples, as shown by stronger staining in the PAs (Fig 2C). Up-regulation of Nox4 expression was also confirmed in hypoxic rats and MCT rats (Fig 2C). Interestingly, the 15-LO inhibitor NDGA reversed the up-regulation of Nox4 in the rat PAs (Fig 2C). Further western blot and real-time PCR studies of PAECs confirmed the dependence of Nox4 gene and protein expression on the 15-LO/15-HETE pathway. Specifically, we observed that Nox4 mRNA and protein levels in PAECs were up-regulated by exogenous 15-HETE and that this effect could be abrogated via inhibition of 15-LO through si15-LO, CDC or NDGA (Fig 2D, 2E and 2G).

As major producers of mROS, mitochondria regulate cellular redox status. To examine the role of mitochondrial Nox4 in response to 15-HETE under hypoxia, we extracted mitochondrial protein and measured mitochondrial Nox4 levels by western blot after the above treatments. The results showed that CDC and NDGA both effectively reduced the up-regulation of mitochondrial Nox4 expression induced by 15-HETE in PAECs (Fig 2F). These data suggest that Nox4, particularly mitochondrial Nox4, is the major player responsible for 15-HETE-mediated ROS regulation.

### The p38 MAPK pathway participates in the up-regulation of Nox4 expression induced by 15-HETE

**Fig 3:**
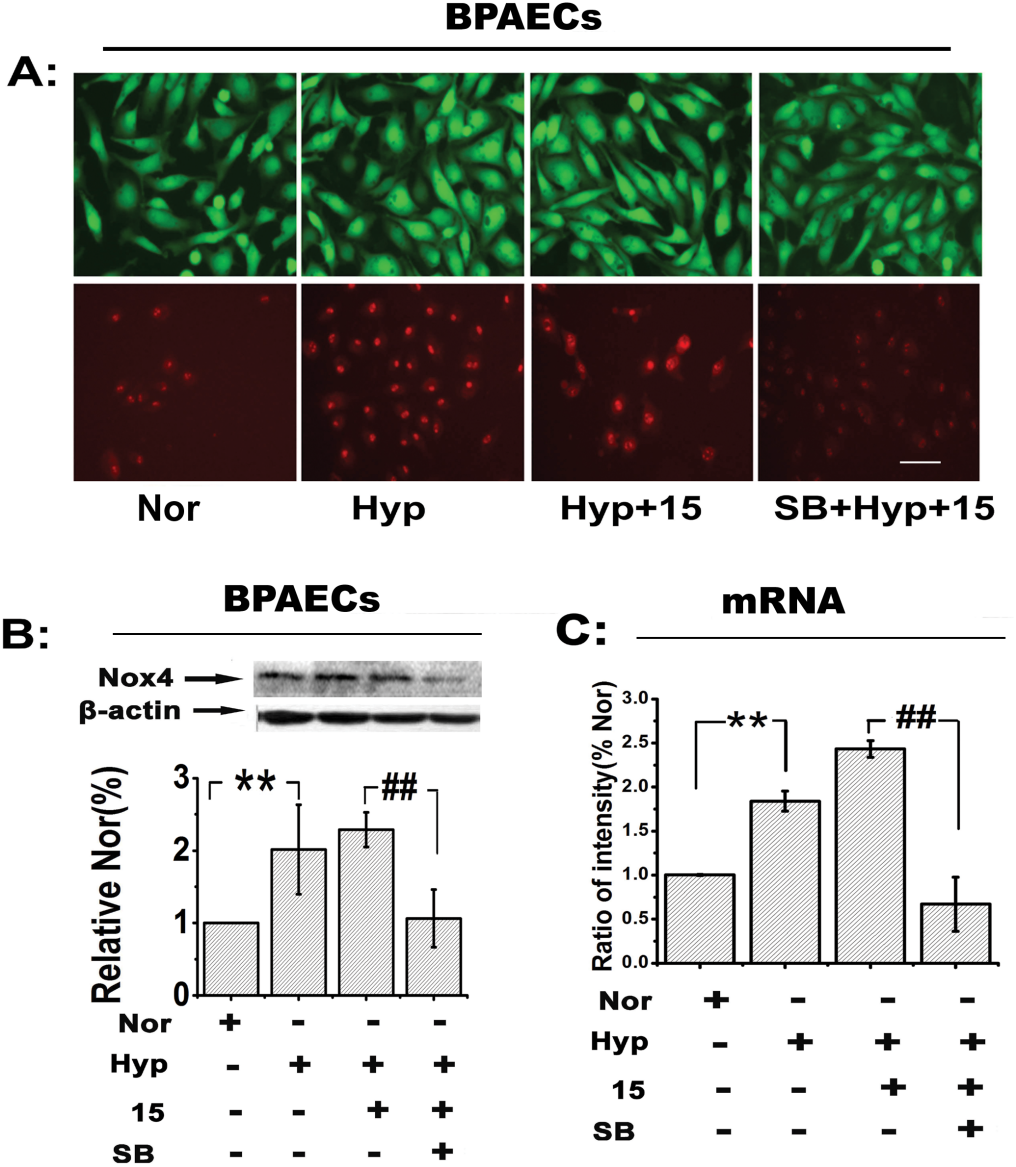
Nox4 expression is up-regulated by 15-HETE, possibly through the p38 MAPK pathway. After pretreatment with SB203580 (10 μmol/L), PAECs were exposed to 15-HETE to measure its effects on **(A)** ROS production (using CM-H2DCFDA or MitoSOX assays; scale bars equal 100 μm) and Nox4 **(B)** protein and **(C)** mRNA expression (western blot and real time-PCR (n = 5)). Nor, normoxia; 15, 15-HETE. Data are presented as the means ± SEM. *p<0.05, **p<0.01 versus normal; **#***p*<0.05, ##*p*<0.01 versus Hyp+15.

Previous studies have reported that p38 MAPK signaling is involved in Nox4 activation [36, 37]. To explore whether the p38 MAPK pathway contributed to the 15-HETE-stimulated induction of Nox4 expression, we first examined the effect of SB203580 (an inhibitor of the p38 MAPK pathway) on ROS production in PAECs. The results showed that SB203580 reduced the elevated ROS production caused by endogenous 15-HETE in PAECs, especially in mitochondria (Fig 3A).

If the hypoxia-induced up-regulation of Nox4 expression were mediated by 15-HETE through the p38 MAPK pathway, inhibition of p38 MAPK signaling should block this effect. To test this hypothesis, western blot and quantitative PCR analyses were performed. The results showed that blockade of the p38 MAPK pathway significantly inhibited the upregulation of Nox4 mRNA and protein stimulated by both exogenous and endogenous 15-HETE (Fig 3B and 3C). These observations indicated that the p38 MAPK pathway is responsible for increasing ROS levels and up-regulating Nox4 expression downstream of 15-HETE under hypoxia in PAECs.

### 15-HETE-induced ROS stimulate bovine PAEC migration and tube formation and promote PASMC proliferation in vitro

**Fig 4:**
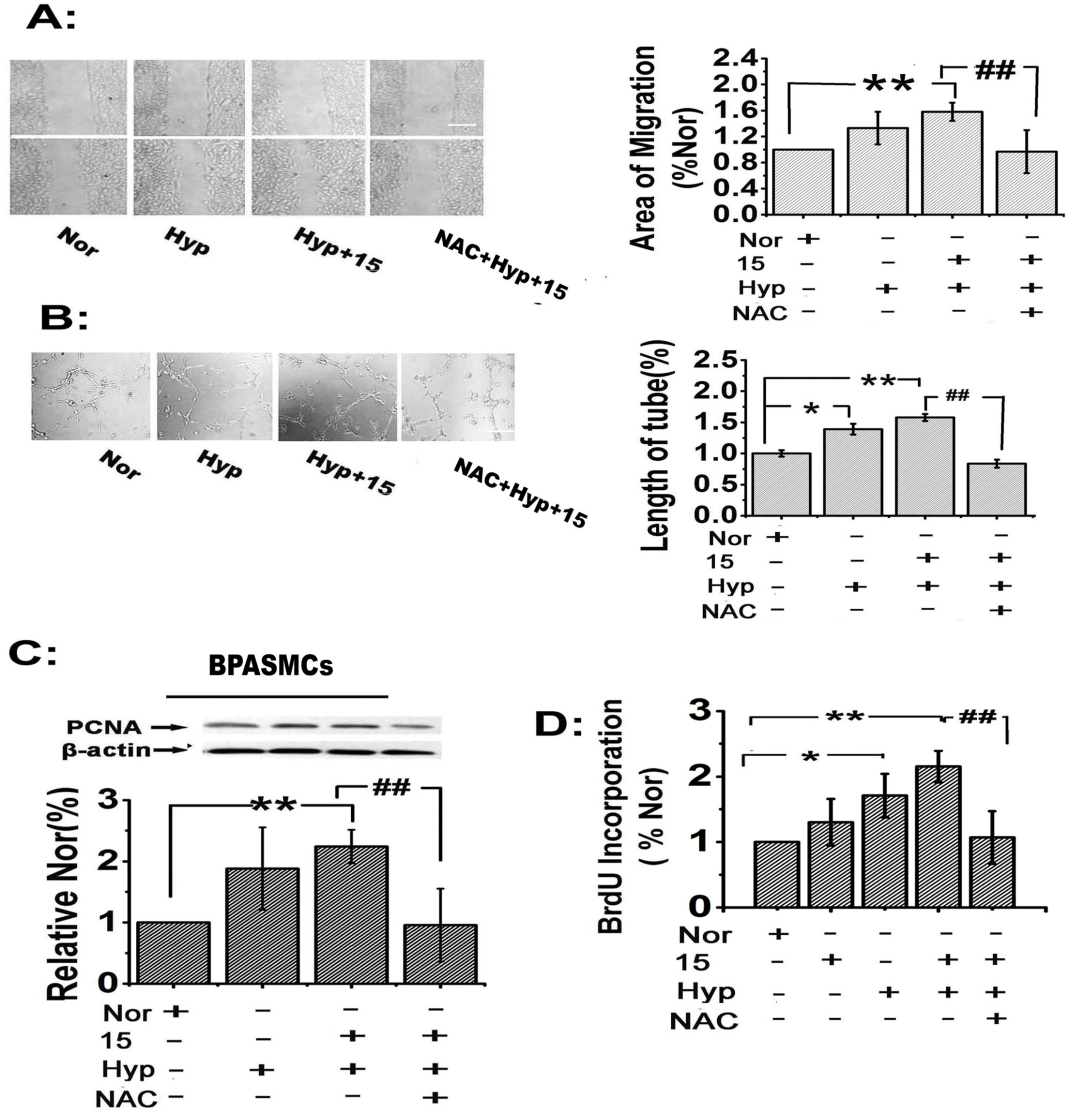
15-HETE-induced ROS promote PAEC migration and tube formation and increase PASMC proliferation. After scavenging ROS with NAC (ROS scavenger), the effects of hypoxia and exogenous 15-HETE on PAEC migration (**A**) were examined by the scratch-wound assay (n = 4). Scale bars indicate 100 μm. Tube formation in PAECs (**B**) was evaluated by the tube formation assay (n = 5). PCNA expression in PASMCs (n = 4) (**C**) was examined by western blot. 5-BrdU incorporation assays were performed to detect DNA synthesis (n = 5). All of the values are expressed as the mean ± SEM. *p<0.05, **p<0.01 versus normal; **#** *p*<0.05, ##*p*<0.01 versus Hyp+15.

To investigate the effects of 15-HETE-induced ROS on PAEC migration and tube formation, we performed tube formation and scratch-wound assays in the presence of NAC (scavenger of ROS; 25 μmol/L). The results showed that both exogenous and endogenous 15-HETE stimulated PAEC migration (Fig 4A) and tube formation (Fig 4B) and that these effects were reversed by scavenging ROS with NAC, indicating that ROS contributed to PAEC migration and tube formation.

To determine the effects of 15-HETE-induced ROS on PASMC proliferation, we compared proliferating cell nuclear antigen levels (PCNA) by western blot analysis and performed a 5-BrdU incorporation assay to assess the population of cells actively synthesizing DNA. We found that exogenous 15-HETE up-regulated the expression of PCNA in PASMCs, while inhibition of endogenous 15-HETE using CDC or NDGA under hypoxic conditions reversed the increase in PCNA levels. More importantly, NAC reversed the up-regulation induced by exogenous 15-HETE, indicating that exogenous 15-HETE had a protective role in cell proliferation (Fig 4C). We also observed that both 15-HETE and hypoxia significantly enhanced 5-BrdU incorporation, but the effect of exogenous 15-HETE was blocked by NAC (Fig 4D). These observations implied that ROS were key factors promoting PASMC proliferation upon exposure to 15-HETE under hypoxia.

### Effect of 15-HETE-induced ROS on cell cycle progression and microtubule dynamic stability in PASMCs

**Fig 5:**
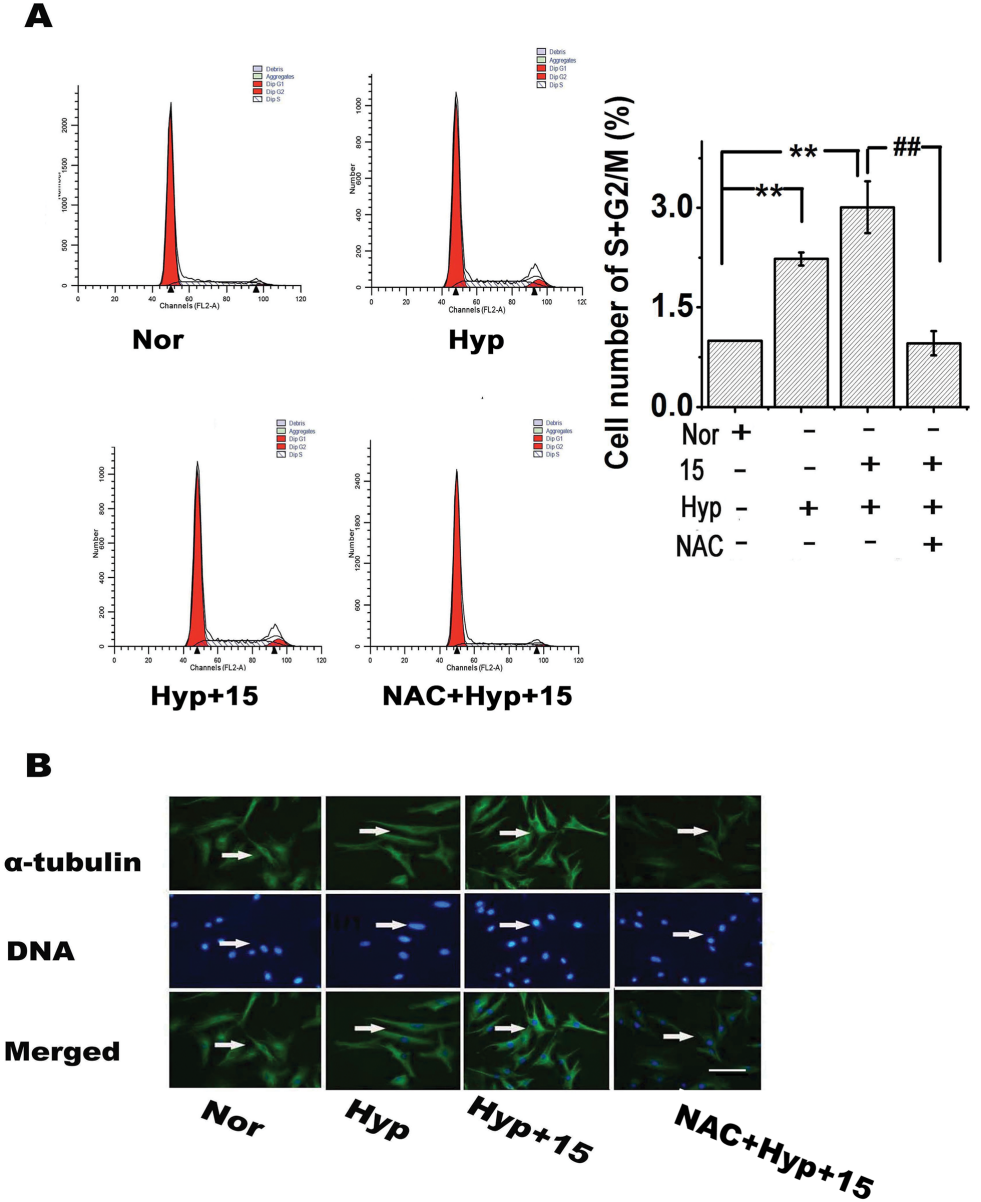
15-HETE-induced ROS promote PASMC cell cycle progression and α-tubulin polymerization in the nucleus. After employing NAC to scavenge ROS in PASMCs, the effects of hypoxia and exogenous 15-HETE on **(A)** the percentages of cells in the S and G2/M phases were analyzed by flow cytometry (n = 3). **(B)** α-tubulin polymerization in the nucleus was detected by immunocytochemistry. Scale bars indicate 100 μm. Data are presented as the means ± SEM; *p<0.05, **p<0.01 versus normal; **#** *p*<0.05, ##*p*<0.01 versus Hyp+15 (n = 3). Nor, normoxia; Hyp, hypoxia; 15, 15-HETE.

To examine whether 15-HETE affected cell cycle progression by increasing ROS levels, we performed cell cycle analysis with flow cytometry and evaluated the organization of the microtubule protein α-tubulin during mitosis by immunofluorescence staining. As shown in Fig 5A, ROS increased the percentages of cells in the S and G2/M phases in the presence of exogenous 15-HETE. Similar to the hypoxia data, the accelerated cell cycle progression was blocked in the presence of NAC, causing more PASMCs to remain in the G0/G1 phase (Fig 5A). We also found that α-tubulin polymerization in the cell nucleus was enhanced under hypoxia or exogenous 15-HETE stimulation compared with the normoxic group, though this microtubule formation was suppressed by pretreatment with NAC (Fig 5B). Together, these data showed that ROS have important roles in 15-HETE-mediated cell cycle progression.

### 15-HETE-induced ROS stimulate PAEC migration and tube formation in vitro via the p38 MAPK pathway

**Fig 6:**
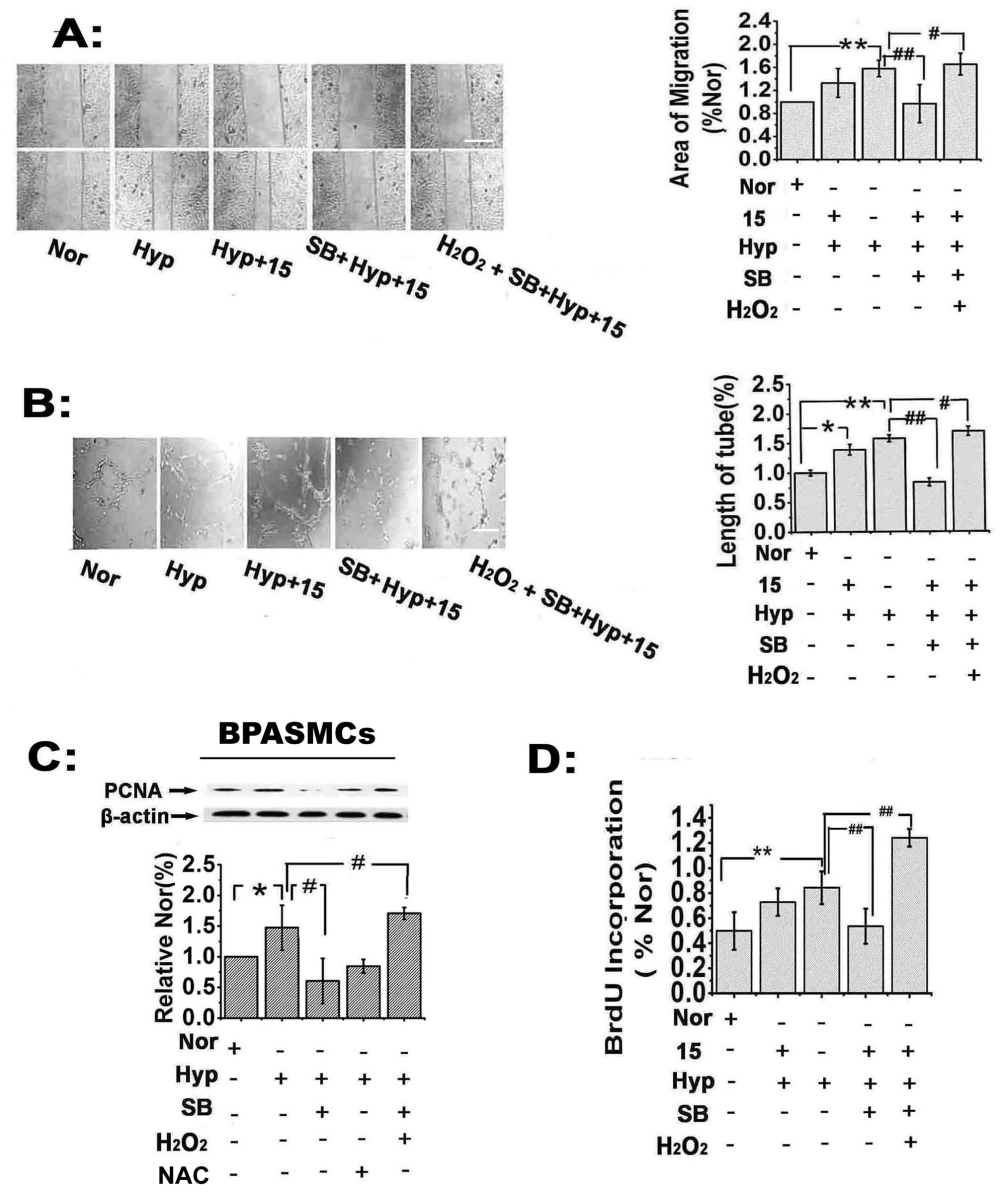
The p38 MAPK pathway contributes to PAEC migration and PASMC proliferation downstream of the ROS induced by 15-HETE. After pretreating cells with H_2_O_2_ (50 μmol/L) plus SB203580 (inhibitor of p38 MAPK signaling) or with SB203580 alone, exogenous 15-HETE was applied in hypoxic conditions, and (**A**) PAEC migration was examined by the scratch-wound assay. Scale bars indicate 100 μm. (**B**) PAEC tube formation was evaluated by the tube formation assay. Scale bars represent 100 μm. (**C**) PCNA expression was examined by western blot (n = 4). (**D**) 5-BrdU incorporation assays were performed to detect DNA synthesis (n = 5). All of the values are expressed as the mean ± SEM; *p<0.05, **p<0.01 versus normal; **#** *p*<0.05, ##*p*<0.01 versus Hyp+15. Nor, normoxia; SB, SB203580; 15, 15-HETE; Hyp, hypoxia.

To explore the role of the p38 MAPK pathway in 15-HETE-ROS-induced PAEC migration and tube formation, we first treated cells with SB203580 to block the p38 MAPK pathway. This significantly inhibited the PAEC migration induced by endogenous and exogenous 15-HETE. However, following pretreatment with H_2_O_2_, the SB203580-mediated inhibition of migration in response to exogenous 15-HETE was relieved. More importantly, 15-HETE-induced PAEC migration was significantly increased (Fig 6A). Similarity, PAEC tube formation was partially blocked by SB203580. After ROS activation by H_2_O_2_, followed by blockade of the p38 MAPK pathway with SB203580, the ability of 15-HETE to induce PAEC tube formation was significantly enhanced (Fig 6B). These data demonstrated that the effects of 15-HETE-regulated ROS on PAEC migration and tube formation were partly dependent on the p38 MAPK pathway.

### 15-HETE-induced ROS stimulate cell cycle progression and promote PASMC proliferation via the p38 MAPK signaling pathway

It is possible that 15-HETE-induced PASMC proliferation requires activation of the p38 MAPK pathway [38, 39]. To evaluate this hypothesis, we first measured PCNA protein levels following blockade of the p38 MAPK pathway. Our results showed that SB203580 treatment reversed the up-regulation of PCNA caused by exogenous and endogenous 15-HETE, whereas H_2_O_2_ (50 μmol/L) abolished this SB203580-mediated inhibition (Fig 6C). The 5-BrdU incorporation assay showed a similar result, in which the effect of 15-HETE on cellular 5-BrdU incorporation was suppressed by SB203580, whereas H_2_O_2_ application significantly blocked the effects of SB203580 (Fig 6D).

**Fig 7:**
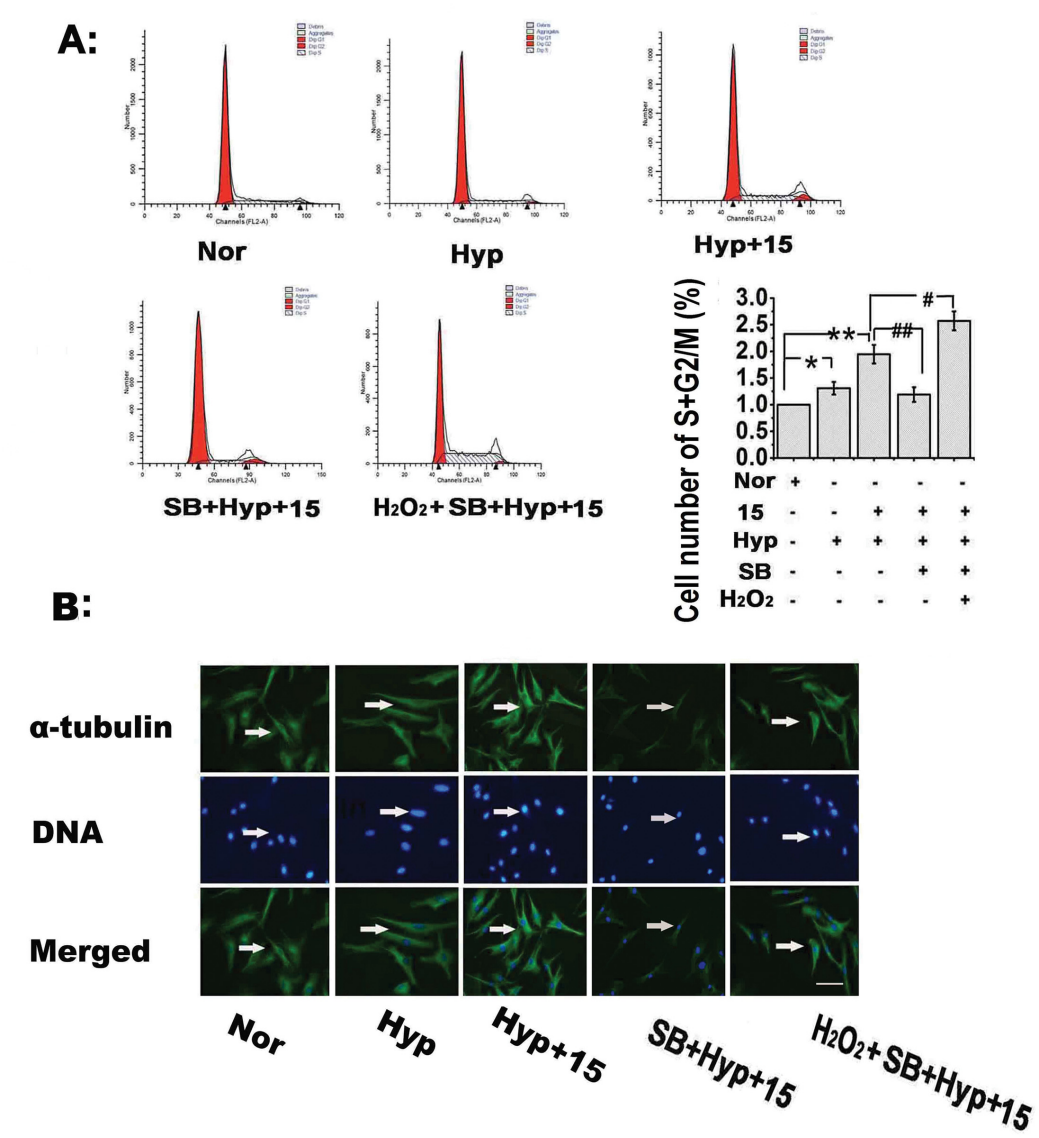
Pharmacological blockade of the p38 MAPK pathway inhibits the PASMC cycle progression and α-tubulin nuclear polymerization induced by 15-HETE via ROS. After adding H_2_O_2_ (50 μmol/L) to activate ROS and then blocking the p38 MAPK pathway with SB203580, or adding SB203580 alone, the effects of endogenous and exogenous 15-HETE on **(A)** PASMC cell numbers in the S and G2/M phases were analyzed by flow cytometry (n = 4). **(B)** α-tubulin polymerization in the nucleus was detected by immunocytochemistry. Scale bars indicate 100 μm. Data are presented as the means ± SEM; *p<0.05, **p<0.01 versus normal; **#** *p*<0.05, ##*p*<0.01 versus Hyp+15. Nor, normoxia; Hyp, hypoxia; 15, 15-HETE.

Next, we blocked p38 signaling with SB203580 to assess whether this pathway also participated in 15-HETE-mediated cell cycle progression. Under hypoxic conditions, greater numbers of cells were promoted to the S and G2/M phases from the G0/G1 phase by endogenous 15-HETE compared with the normoxic group, and this effect was reversed by SB203580. However, after pretreating cells with H_2_O_2_, followed by blockade of the p38 MAPK pathway with SB203580, exogenous 15-HETE significantly affected cell cycle activity (Fig 7A). We also found that SB203580 suppressed the exogenous 15-HETE-stimulated α-tubulin polymerization in the cell nucleus under hypoxia; this effect was abolished by pretreatment with H_2_O_2_ (Fig 7B). Taken together, these data indicated that the p38 MAPK pathway contributed to the PASMC proliferation induced by 15-HETE-regulated ROS in hypoxic conditions.

## Discussion

**Fig 8:**
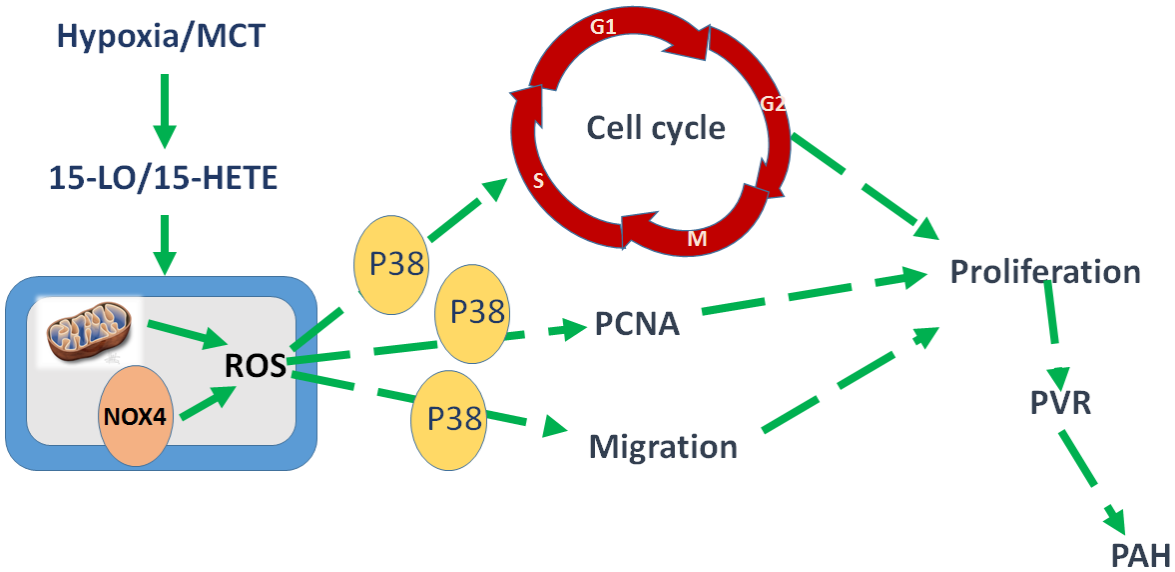
A proposed schematic links between 15-LO/15-HETE, ROS, and proliferation in PVR under hypoxia. The ROS induced by 15-HETE are generated in the mitochondria and by Nox4. These ROS stimulate migration, cell cycle progression, and proliferation via the p38 MAPK pathway. This process triggers pulmonary vascular remodeling to cope with hypoxic pulmonary hypertension.

It is still controversial in the field whether hypoxia increases or decreases ROS in PAs [40–42]. Most studies agree that HPH is related to the regulation of ROS production, leading to PVR and pulmonary hypertension [13, 43–47], although the cellular and molecular mechanisms remain unclear. One major finding of our study was that hypoxia enhances ROS production in PAECs through the 15-LO/15-HETE pathway. We also demonstrated that the major cellular sources of ROS induced by 15-HETE under hypoxia in PAECs were mitochondria and vascular Nox4 oxidase. Furthermore, 15-HETE-induced ROS are key molecules that function through the p38 MAPK pathway to promote cell cycle progression and PASMC proliferation under hypoxia, leading to PVR. Specifically, we provide evidence to demonstrate the role of 15-HETE-mediated ROS activation in response to hypoxia, leading to hypoxia-induced PVR and PH (Fig 8).

Various studies have reported that ROS are key mediators produced in response to chronic hypoxia that can promote PH by increasing vascular remodeling. However, relatively little is known about the molecular mechanisms of ROS generation in hypoxic PVR. Previous studies, including work from our laboratory, have shown that 15-HETE, the metabolite of 15-LO, has a crucial role in hypoxia-induced PVR [42, 47, 48]. Thus, we sought to address whether hypoxia regulates the generation of ROS through the 15-LO/15-HETE pathway and whether the ROS regulated by 15-HETE in the hypoxic PVR condition contribute to PH development. Our data showed that hypoxia increased the production of ROS, consistent with previous reports [43–47]. Both exogenous and endogenous 15-HETE promoted the generation of ROS, and this response was blocked in the presence of a chemical inhibitor of 15-LO or si15-LO. These data indicate that 15-LO/15-HETE contributes to the increased generation of ROS under hypoxia, confirming our hypothesis.

It is well known that mitochondria serve as major cellular sources of intracellular O_2_^.-^, although their contribution to ROS production under hypoxic conditions is poorly understood [49, 50]. One study indicated that mitochondrial O_2_^.-^contributes to sustained (120 minutes) hypoxic pulmonary vasoconstriction in isolated perfused rabbit lungs. However, where the ROS were generated was unknown. By using NADPH oxidase and mitochondrial inhibitors, we found that the sources of the ROS generated by 15-HETE differed between PAECs and PASMCs. Interestingly, mitochondria-derived ROS appear to be the predominant form induced by 15-HETE in PASMCs; conversely, in PAECs, the main sources were the mitochondrial respiratory chain and Nox4, which is a unique Nox isoform that has also been described as an oxygen sensor [51–54]. In the vascular system, modulation of signaling events by endothelial cells (ECs) is essential to the ability of this cell monolayer to regulate the underlying VSMCs. For this reason, we mainly focused on examining the quantitative changes in ROS in PAECs as well as the series of cell responses promoted by 15-HETE.

To explore the cellular mechanisms of this process, we evaluated the effect of 15-HETE on Nox4 gene and protein expression. We found that Nox4 was overexpressed in remodeled PAs from PH patients and hypoxic PH rats as well as in rats injected with monocrotaline. Nox4 protein levels in PAECs and their mitochondria were also up-regulatedby treatment with 15-HETE, whereas this effect was attenuated by the administration of a 15-LO inhibitor or si15-LO. Similar results were obtained for Nox4 gene expression levels by real-time PCR. One possible explanation for this finding is that the 15-LO/15-HETE pathway may contribute to hypoxia-induced ROS through its effects on mitochondria and Nox4. These results also revealed that the sources of ROS stimulated by 15-HETE in PAECs were the mitochondrial respiratory chain and Nox4.

Accumulating evidence has shown that hypoxia can stimulate PASMC proliferation, which is a key component of PVR [47, 52, 55, 56]. Consistent with previous reports [57], our results showed that exogenous and endogenous 15-HETE activated α-tubulin polymerization in the nucleus, increased the numbers of PASMCs in the S and G2/M phases, induced PAEC tube formation and migration in vitro, and up-regulated PCNA expression in PASMCs. However, these effects were blocked after scavenging ROS by NAC. These data provided direct evidence that 15-HETE promotes PASMC proliferation by regulating ROS.

Many studies have linked the activation of p38 MAPK under hypoxia to the resulting pulmonary vascular remodeling and increased cell proliferation. Our laboratory previously reported that 15-LO/15-HETE mediated vascular adventitia fibrosis via the p38 MAPK pathway [22, 58]. In this study, we also investigated the role of the p38 pathway in 15-HETE-mediated NOX_4_ overexpression and PASMC cell proliferation. We used an inhibitor of the p38 MAPK pathway and hydrogen peroxide (H_2_O_2_) as an activator of ROS, which can stimulate vascular SMC proliferation [59]. We found that inhibition of the p38 MAPK pathway could reverse the overexpression of Nox4 induced by 15-HETE. Interestingly, the 15-HETE-mediated overexpression of Nox4 expression was significantly enhanced by both H_2_O_2_ and p38 MAPK inhibitor treatment. In addition, our results also showed that blockade of the p38 MAPK pathway in PASMCs inhibited PCNA expression, decreased the proportions of cells in the S and G2/M phases, and reduced PAEC tube formation and migration in vitro. All of these effects were reversed upon H_2_O_2_ treatment, as a result of ROS production. Thus, these observations provided a new molecular mechanism and showed that 15-HETE-induced ROS modulate PA cell proliferation via the p38 MAPK pathway, although other downstream effectors in this process remain to be identified. In addition, further study is necessary to evaluate whether the cellular damage induced by excessive ROS further promoted ROS production (ROS-induced ROS release, RIRR), which could be the actual cause of the proliferation. Further studies in this important area will aid in understanding the protein-protein interaction network involved in the regulation of ROS induced by 15-HETE in PH.

## Conclusion

The data in the present study suggested that both mitochondria and Nox4 play important roles in the process of 15-HETE-mediated induction of ROS under hypoxia. Moreover, the ROS induced by 15-HETE under hypoxia affected pulmonary cell proliferation and cell cycle progression, leading to PA remodeling and pulmonary hypertension. The p38 MAPK pathway was also involved in this process. These findings provide new evidence that ROS generated by mitochondria and Nox4 were involved in 15-HETE-mediated hypoxic PVR. Although our results have elaborated the mechanism of the key role of ROS in the pathology of HPH induced by 15-HETE and may provide novel therapeutic insight into the treatment of PH, we were not able to evaluate dynamic physiological levels of ROS; therefore, treatments aimed at effective scavenging of ROS in PH remain an important challenge.
